# Mutational spectrum and phenotypic variability of Duchenne muscular dystrophy and related disorders in a Bangladeshi population

**DOI:** 10.1038/s41598-023-48982-w

**Published:** 2023-12-06

**Authors:** Shaoli Sarker, Tamannyat Binte Eshaque, Anjana Soorajkumar, Nasna Nassir, Binte Zehra, Shayla Imam Kanta, Md Atikur Rahaman, Amirul Islam, Shimu Akter, Mohammad Kawsar Ali, Rabeya Akter Mim, K. M. Furkan Uddin, Mohammod Shah Jahan Chowdhury, Nusrat Shams, Md. Abdul Baqui, Elaine T. Lim, Hosneara Akter, Marc Woodbury-Smith, Mohammed Uddin

**Affiliations:** 1 Genetics and Genomic Medicine Centre (GGMC), NeuroGen Healthcare, Dhaka, Bangladesh; 2 Bangladesh Shishu Hospital and Institute, Dhaka, Bangladesh; 3 Center for Applied and Translational Genomics (CATG), Mohammed Bin Rashid University of Medicine and Health Sciences, Dubai, UAE; 4 GenomeArc Inc., Mississauga, Ontario Canada; 5 Department of Biochemistry, Holy Family Red Crescent Medical College and Hospital, Dhaka, Bangladesh; 6 Department of Genomics and Computational Biology, University of Massachusetts Chan Medical School, Worcester, MA 01605 USA; 7 Department of Molecular, Cell and Cancer Biology, University of Massachusetts Chan Medical School, Worcester, MA 01605 USA; 8 Biosciences Institute, Newcastle University, Newcastle upon Tyne, NE2 4HH UK

**Keywords:** Diseases of the nervous system, Neurological disorders, Behavioural genetics, Clinical genetics, Cytogenetics, Genotype, Medical genetics, Mutation, Neurodevelopmental disorders, Development, Diseases

## Abstract

Duchenne muscular dystrophy (DMD) is a severe rare neuromuscular disorder caused by mutations in the X-linked dystrophin gene. Several mutations have been identified, yet the full mutational spectrum, and their phenotypic consequences, will require genotyping across different populations. To this end, we undertook the first detailed genotype and phenotype characterization of DMD in the Bangladeshi population. We investigated the rare mutational and phenotypic spectrum of the *DMD* gene in 36 DMD-suspected Bangladeshi participants using an economically affordable diagnostic strategy involving initial screening for exonic deletions in the *DMD* gene via multiplex PCR, followed by testing PCR-negative patients for mutations using whole exome sequencing. The deletion mapping identified two critical *DMD* gene hotspot regions (near proximal and distal ends, spanning exons 8–17 and exons 45–53, respectively) that comprised 95% (21/22) of the deletions for this population cohort. From our exome analysis, we detected two novel pathogenic hemizygous mutations in exons 21 and 42 of the *DMD* gene, and novel pathogenic recessive and loss of function variants in four additional genes: *SGCD*, *DYSF*, *COL6A3*, and *DOK7*. Our phenotypic analysis showed that DMD suspected participants presented diverse phenotypes according to the location of the mutation and which gene was impacted. Our study provides ethnicity specific new insights into both clinical and genetic aspects of DMD.

## Introduction

Duchenne muscular dystrophy (DMD, OMIM: 310200) is a lethal pediatric muscle disorder that affects males with an incidence of 1 in 3500 to 1 in 5000 live births^1^. Mutations in the *DMD* gene (HGNC: 2928), the largest human gene located on Xp21, the short arm of the X-chromosome, are responsible for the early onset of this disease^2^. This gene is composed of 79 exons, which encode dystrophin, a cytoskeletal protein consisting of 3685 amino acids. Dystrophin plays a critical role in anchoring the cytoskeleton to the plasma membrane^3,4^. Patients with DMD experience severe and progressive skeletal, respiratory, and cardiac muscle weakness, resulting in delayed physical milestones, often leading to non-ambulatory and wheelchair dependence by early adolescence. Clinical manifestations are also accompanied by various developmental disorders, including speech delay, learning disability, and/or cognitive impairment^1,5,6^. Hence, efficacious disease management demands the precise and early detection of DMD that can accelerate earlier intervention, comprehensive genetic counseling, and suitable assignment to clinical trials.

Rare disorders often manifest a spectrum of phenotypes^7–9^ and harbour numerous types of genetic variants that are strongly associated with disease pathophysiology^7,10^. Genetic studies on DMD cohorts demonstrate that single or multiple exonic deletions/duplications in the dystrophin gene are the most common type of mutation worldwide, with higher mutation frequencies observed within exons 44–55, resulting in prematurely cleaved proteins^11–13^. However, Bladen et al. (2015) studied a large DMD cohort comprising 7000 patients and reported two regions ranging from exon 2–20 and exon 45–55 as hot spots for *DMD* deletions^11^. Although no true/complete population-based systematic assessments have been performed to date, most mutation surveys indicate that the spectrum of *DMD* gene mutations comprises 79% of the patients identified with large mutations (more than one exon affected), of which 68% account for large deletions and 11% for large duplications. The remaining 21% of patients carry small mutations, out of which half are nonsense mutations, while small deletions, small insertions, and splice site mutations represent 5%, 2%, and 3% of the total patient population, respectively^14–16^.

Accurate DNA diagnostic analysis is crucial for patients since it is important for optimal care and family planning, but it also provides information on eligibility for mutation-specific treatments^17–19^. Most underdeveloped or poor countries lack proper molecular diagnosis guidelines for DMD-suspected patients. Currently, several diagnostic techniques are available for identifying deletion patterns such as Multiplex Ligation-dependent Probe Amplification (MLPA), Next Generation Sequencing (NGS), or a combination of multiplex PCR and Southern blotting. For the identification of large deletions, multiplex PCR is generally adopted as the first-step diagnosis to allow the detection of approximately 98% of deletions, which accounts for 65% of all mutations. However, this technique is ineffective in detecting duplications, defining deletion boundaries, or determining reading frame disruption^15^. Furthermore, when negative results are obtained from PCR, NGS analysis can be utilized to uncover mutations, including point mutations, small deletions, and duplications or insertions in exons, promoters, or known intronic mutations^8,20,21^. A combination of hotspot deletion screening and exome sequencing seems an economically viable option for developing countries.

In this study, we performed clinical and molecular diagnoses in 36 DMD-suspected male participants adopting the multiplex *DMD* deletion hotspot test and exome sequencing. Our objective was to determine the nature of genetic variants in the *DMD* gene in this population and to capture the phenotypic characteristics and mutational landscape for DMD-suspected patients from Bangladesh, a genetically underrepresented population with high consanguinity. To our knowledge, this is the first genetic analysis in the Bangladeshi population to investigate the DMD/ related myopathies using a combination of PCR and NGS, enabling cost-effective and accurate diagnoses. We hypothesized that this combination would offer high diagnostic yield both in relation to the *DMD* gene but also other genes of potential etiological significance and thereby offer a cost-effective approach to genetic diagnosis in DMD patients in developing countries. facilitating economically viable and accurate diagnosis and assisting clinicians in early intervention and timely therapeutic management.

## Methods

### Subject recruitment and clinical study

Thirty-six unrelated male individuals clinically diagnosed with DMD from multiple tertiary hospitals in Bangladesh were referred to NeuroGen Healthcare for genetic evaluation from September 2020 to December 2022. All patients presented with symptoms that included the presence of positive Gower sign, high CPK level, lower limb weakness, inability to use stairs, increased walking difficulties, and calf hypertrophy. Genetic analysis was performed based on the approval of parents through signed written informed consent. The study protocol was approved by the Institutional Review Board of Holy Family Red Crescent Medical College and Hospital, Dhaka, Bangladesh. All methods in this study were performed in accordance with the relevant guidelines and regulations.

### DNA extraction

Blood samples were collected from participants in EDTA vacutainers. Genomic DNA was then extracted using ReliaPrep™ Blood gDNA isolation kit (Promega, USA) according to manufacturer instructions. The quality and quantity of DNA were determined using NanoPhotometer C40 (Implan, Germany) and on 0.8% agarose gel. DNA was stored at -20˚C until further use.

### Multiplex PCR

Multiplex PCR analysis was performed using a panel comprised of 26 target exons^22–24^. In this panel, a total 23 pairs of primers were newly designed using Primer 3 plus software, IDT, and UCSC Genome Browser (Supplementary Table 1). Additionally, we incorporated three pairs of primers from the study by Chamberlain et al. in 1988^25^.

Multiplex PCR was conducted in 5 sets using GoTaq® Hot Start Colorless Master Mix (Promega, USA) to amplify all 26 amplicons. PCR products were separated on 2% agarose gel and were imaged using a gel documentation system.

### Whole exome sequencing

For whole exome sequencing (WES), we employed the Twist NGS Target Enrichment workflow, a solution-based system that utilizes ultra-long 120-mer biotinylated cDNA baits. These baits were used to selectively capture the regions of interest, effectively enriching them from the NGS genomic fragment library. Our workflow included the Twist Core Exome plus RefSeq Panel (hg38) comprising a 36.7 Mb target region. Library preparation and target enrichment followed the Twist Bioscience library protocol, and sequencing was carried out using the Illumina NovaSeq 6000 system with 150 bp paired-end sequencing. The average coverage was 100X on raw data and > 70X on target. Detailed mapping information for each sample can be found in Supplementary Table 5. Reads were mapped to the human reference genome GRCh38/UCSC hg38 with Burrows–Wheeler Aligner (BWA)^25^ and the Genome Analysis Toolkit (GATK 4.0.11.0)^26^. ANNOVAR (2018 Apr 16 version) was used for variant functional annotation. Further, we used another genomic variant annotation tool, Horizon version 1, GenomeArc Inc, that predicts pathogenicity and allows custom clinical genetic mutation database integration with other large-scale publicly available databases (i.e., gnomAD, ClinVar, pLI). Variant classification analysis was conducted based on the American College of Medical Genetics (ACMG) guidelines^27^.

### CNV analysis from WES data

CNVkit^28^ was used to analyse the CNV in 8 patients who did not present causative variants in the *DMD* gene. To establish a suitable reference for our analysis, we employed the hg38 assembly and a male control sample which had no muscle related abnormalities. WES data for this control sample did not reveal any short nucleotide variations or gross deletions within the exons of the *DMD* gene.

### Variant validation using Sanger sequencing

The variants identified from WES were validated by Sanger sequencing using standard protocols. Validation primers (Supplementary Table 2A) were designed using Primer 3 plus, IDT, and UCSC Genome Browser. Primer and PCR conditions are included (Supplementary Table 2B). The PCR products were run on 2.0% agarose gel and the products were then purified using the Wizard® SV Gel and PCR Clean-Up System (Promega, USA) according to the manufacturer’s instructions. Cycle sequencing was performed using purified PCR products as a template and BigDye® Terminator v3.1 (Applied Biosystem, USA). Bidirectional Sanger sequencing was performed using 3500 DNA Analyzer (Applied Biosystem, USA) to determine the sequence. Sanger sequencing data were analyzed using Sequence Scanner v2.0 (Applied Biosystem, USA).

## Results

### Clinical diagnosis and phenotypic characterization of DMD-suspected participants

**Figure 1:**
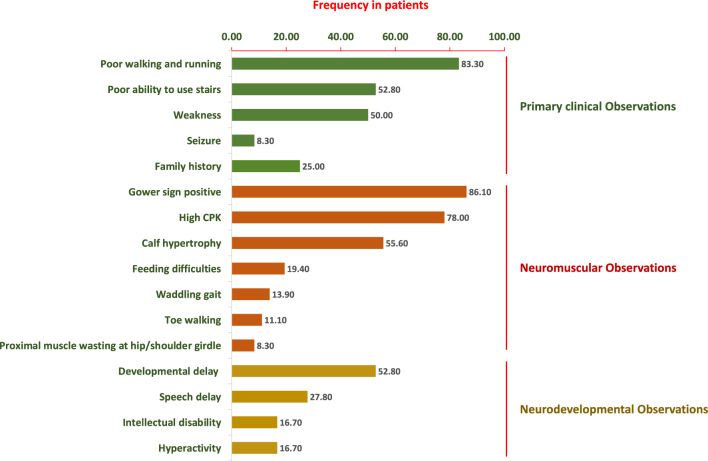
Frequency of clinical characteristics in 36 DMD-suspected patients in the Bangladeshi cohort.

It is recognised that DMD symptoms in an individual appear before 5 years of age with high serum creatine kinase and a lack of dystrophin protein in muscle biopsy^29^. However, at the time of genetic testing, the mean age of the 36 clinically diagnosed DMD participants in our study cohort was 7.1 years (range from 1.5 years to 22 years). Further, data on 24 of the participants indicate the average age of onset of symptoms was as early as 4.62 years. General physical examination demonstrated that participants exhibited a wide range of motor abnormalities, including Gowers' sign in 31 patients (86%), poor walking and running ability in 30 patients (83%), and hypertrophy of calf muscles in 20 patients (56%). In the cohort, only 4 (11%) children were found to walk on their toes, while 19 (53%) cases had a poor ability to use stairs, 18 (50%) had muscle weakness, 5 (14%) had waddling feet and abnormal gait, 7 (19%) had feeding difficulties and 3 (8%) cases were presented with skinny legs and arms. Nineteen (53%) participants were reported to have developmental delays, 10 participants (29%) had a speech delay at the time of diagnosis, 6 (17%) had an intellectual disability and 6 (17%) participants had motor hyperactivity. Biochemical analysis in the cohort showed that 28 (78%) participants had high serum Creatine Phosphokinase (CPK) levels ranging from 453 to 36,210 U/L (mean, 13,451.96 U/L). In addition, 9 out of 36 participants (25%) had a family history of muscular dystrophy and 3 (9%) participants had experienced at least one seizure. The clinical phenotypes of the DMD participants in our study are presented in Fig. 1, while detailed clinical and laboratory data are summarized in Supplementary Table 3.

### *DMD* deletion hotspot and pathogenic variants

**Figure 2:**
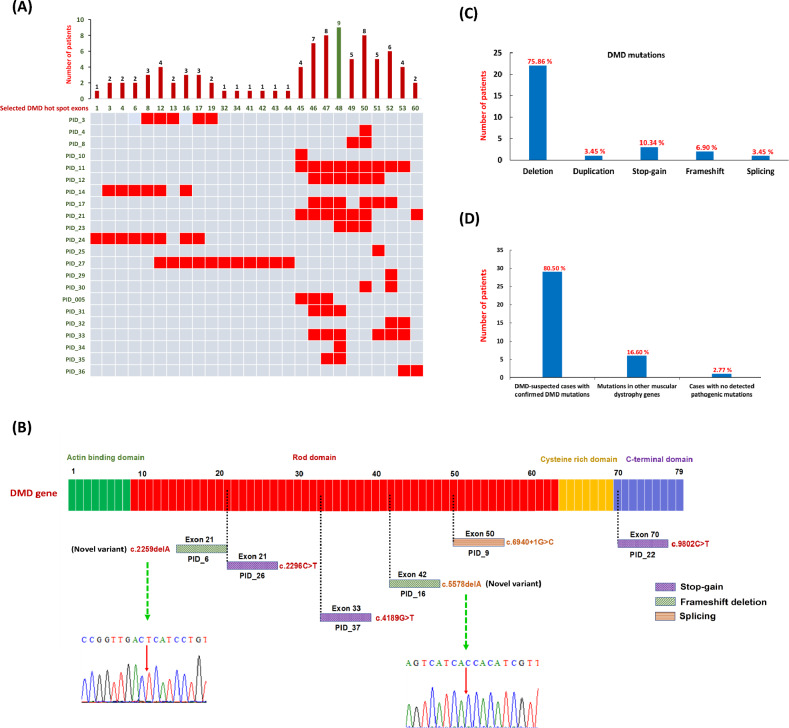
Mutation landscape in 36 DMD-suspected patients in the Bangladeshi cohort. **(A)** Mutation spectrum of *DMD* gene in 26 selected exons. The plot indicates the number of patients showing deletion mutations in the 36 DMD cases. The data showed that deletion of exon 48 is frequently seen in DMD-affected individuals. It was found to be deleted in 9 out of 22 positive test results. The heatmap demonstrates the deletion mutation in 22 PCR-positive patients in the *DMD* gene exons. The rows and columns represent the patient ID and exons respectively. The squares highlighted in red are the exons in which deletions are reported in each patient. **(B)** Schematic presentation of the point mutation in the *DMD* gene by WES. The Dystrophin gene contains 79 exons spanning the N-terminal actin-binding domain, central rod domain, Cysteine-rich domain, and C-terminal domain. Green dotted lines indicate the novel mutations. *DMD* gene mutations were detected in six patients. **(C)** Percentage breakdown of mutation types in DMD-positive cases. (D) Percentile distribution of confirmed *DMD* gene mutations, other muscular dystrophy genes, and cases with no pathogenic mutations.

Our study aimed to diagnose the deletion mutation pattern in 36 DMD-suspected participants. We observed that out of 36 unrelated clinically diagnosed DMD males, 22 patients (61%) were found positive, and 14 patients (39%) were negative for *DMD* single or multiple exon deletions assessed by multiplex PCR (Supplementary Fig. 1). A total of 88 exons were deleted in 22 patients, while the average number of exons deleted per participant was 4. Furthermore, out of 22 participants with deletions, only 5 participants (22.7%) carried a single exon deletion (Fig. 2A), whereas the other 17 participants (77.2%) had multiple deleted exons spanning the major hot spot regions in the *DMD* gene (Fig. 2A). However, 14 participants with negative multiplex PCR results did not possess any clinically relevant deletion in the selected hotspot regions of the gene (Supplementary Table 1).

A comparative analysis of the deleted exonic regions in the *DMD* gene among positive cases revealed that most deletions were found clustered in the two critical hotspot regions of the *DMD* gene (i.e., near proximal and distal ends, spanning exons 8–17 and exons 45–53, respectively) comprised of 95% (21/22) of all deleted cases. Precisely, 9 positive participants had a deletion of exon 48 near the central part of the *DMD* gene, while exon 47 and exon 50 were found missing in another 8 participants with concurrent deletion of exons 46 and 52 in 7 and 6 participants respectively. Deletions near the proximal end (exons 8–17) were comparatively less frequent; however, within this region, exon 12 was found to be missing in a total of 4 participants. Additionally, less frequent deletions in exons 32, 34, 41, 42, 43, and 44 were observed among the participants in the study cohort.

During genetic testing, participant samples that were negative for deletion mutations in the *DMD* gene were subjected to WES analysis. The results indicate that out of 14 participants, 13 carried pathogenic or clinically relevant mutations in *DMD* and/or its associated muscular dystrophy genes (Table 1). Figure 2B illustrates the point mutations identified by WES in DMD-positive cases. Five individuals, *i.e.,* PID_37, PID_26, PID_22, PID_9, and PID_6, were found to possess point mutations only in the *DMD* gene (Table 1). In individuals PID_37, PID_26, and PID_22, we identified a hemizygous stop-gain mutation (c.4189G>T) in exon 33, another hemizygous stop-gain mutation (c.2296C>T) in exon 21, and a hemizygous stop-gain mutation (c.9802C>T) in exon 70 of the *DMD* gene respectively. On the other hand, PID_9 was found to have a hemizygous splicing mutation (c.6940+1G>C) in exon 50, and PID_6 showed a hemizygous frameshift deletion (c.2259delA) in exon 21 of the *DMD* gene. The mutation types identified in the *DMD* gene by WES are shown in Fig. 2C.

**Table 1:** Point mutations identified by whole exome sequencing in 14 analyzed participants.

ID	Chr	Gene name	Exon	Nucleotide change	Protein change	Variant type	Zygosity	ACMG classification
PID_37	ChrX	*DMD*	Exon 33	NM_004010 c.4189G>T	p.Glu1397Ter	Stopgain	Hom	Pathogenic
PID_28	ChrX	*DMD*	Duplication of Exon 45 & 47					Pathogenic
PID_26	ChrX	*DMD*	Exon 21	NM_004010 c.2296C>T	p.Arg766Ter	Stopgain	Hom	Pathogenic
PID_22	ChrX	*DMD*	Exon 70	NM_004010 c.9802C>T	p.Arg3268Ter	Stopgain	Hom	Pathogenic
PID_6	ChrX	*DMD*	Exon 21	NM_004010 c.2259delA	p.Val754Ser fs*14 (novel)	Frameshift deletion	Hom	Pathogenic
PID_9	ChrX	*DMD*	Exon 50	NM_004010 c.6940+1G>C		Splicing	Hom	Pathogenic
PID_16	ChrX	*DMD*	Exon 42	NM_004010 c.5578delA	p.Met1860Trp fs*2 (novel)	Frameshift deletion	Hom	Pathogenic
	Chr22	*LZTR1*	Exon 8	NM_006767 c.791+1G>A		Splicing	Het	Pathogenic
PID_19	Chr5	*SGCD*	Exon 4	NM_172244 c.204_207del	p.Asn69* (novel)	Stopgain	Hom	Pathogenic
PID_13	Chr15	*WDR72*	Exon 8	NM_182758 c.764_768del	p.Gly255Val fs*40	Frameshift deletion	Hom	Pathogenic
	ChrX	*PHEX*	Exon 1	NM_001282754 c.10G>C	p.Glu4Gln	Missense	Hom	Pathogenic
PID_5	Chr2	*DYSF*	Exon 13	NM_001130987 c.1254_1255insCGGGCCGAGGACTTGCCGCAGAGTG	p.Met426Ser fs*26 (novel)	Frameshift insertion	Het	Pathogenic
	Chr2	*DYSF*	Exon 55	NM_001130987 c.6313G>A	p.Ala2105Thr	Missense	Het	Pathogenic
PID_15	Chr15	*MAGEL2*	Exon 1	NM_019066 c.1655C>G	p.Pro552Arg	Missense	Het	VUS
	Chr2	*ZEB2*	Exon 8	NM_014795 c.2234C>A	p.Ala745Glu	Missense	Het	VUS
	ChrX	*ABCD1*	Exon 10	NM_000033 c.2188C>T	p.Pro730Ser	Missense	Hom	VUS
PID_155	Chr14	*MYH7*	Exon 31	NM_000257 c.4259G>A	p.Arg1420Gln	Missense	Het	Pathogenic
	Chr2	*COL6A3*	Exon 16	NM_004369 c.6210+1G>A		Splicing	Het	Pathogenic
PID_123	Chr2	*COL6A3*	Exon 9	NM_004369 c.3958_3959insGTGT	p.S1320Cfs*15 (novel)	Frameshift insertion	Het	Pathogenic
PID_108	Chr4	*DOK7*	Exon 7	NM_173660 c.1134delG	p.A380Pfs*76 (novel)	Frameshift deletion	Hom	Pathogenic

### Genetic variants in *DMD* negative patients

**Figure 3:**
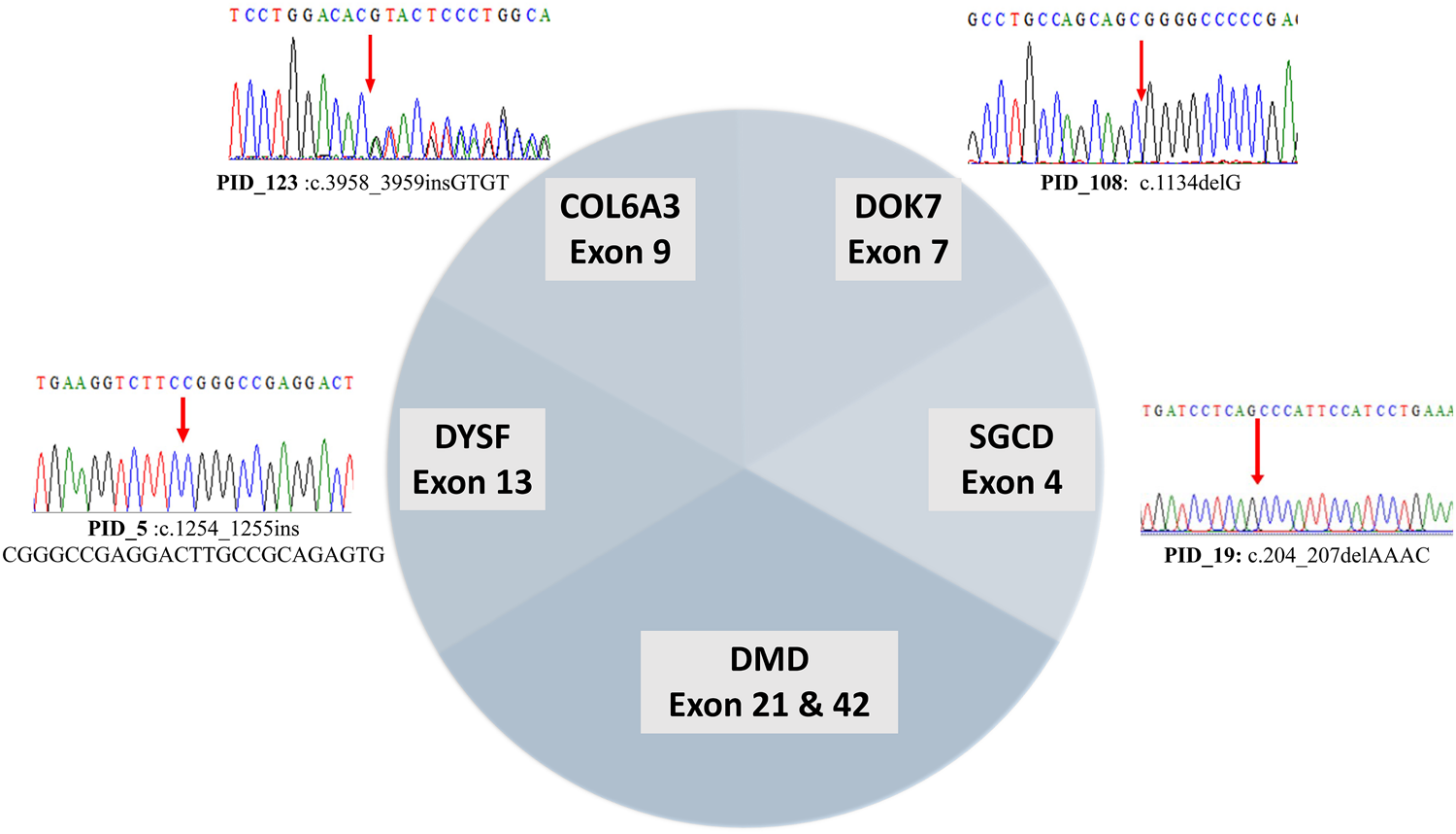
Representation of six novel mutations (with exon numbers) and chromatogram images showing validation of novel mutations in other myopathy/ muscular dystrophy genes detected in our WES analysis.

We have conducted exome sequencing for DMD multiplex PCR negative participants to identify novel mutations and genes related to musculoskeletal diseases. Interestingly, the other 6 cases, PID_19, PID_13, PID_5, PID_155, PID_123, and PID_108, presented novel variants (Sanger verified) in other myopathy—associated genes (Table 1, Fig. 3). This includes a homozygous stop-gain deletion (c.204_207del) in exon 4 of the *SGCD* gene (HGNC: 10807) in PID_19, homozygous frameshift deletion (c.764_768del) in exon 8 of the *WDR72* gene (HGNC: 26790), and a homozygous missense mutation (c.10G>C) in exon 1 of the *PHEX* gene (HGNC: 8918) in PID_13, heterozygous frameshift insertion (c.1254_1255insCGGGCCGAGGACTTGCCGCAGAGTG) and a heterozygous missense mutation (c.6313G>A) in exon 13 of *DYSF* (HGNC: 3097) in PID_5, a heterozygous missense mutation (c.4259G>A) in the exon 31 of *MYH7* (HGNC: 7577) and heterozygous splicing mutation (c.6210+1G>A) in the exon 16 of *COL6A3* (HGNC: 2213) in PID_155 and lastly, another heterozygous frameshift insertion (c.3958_3959insGTGT) in exon 9 of *COL6A3* and homozygous frameshift deletion (c.1134delG) in the exon 7 of *DOK7* (HGNC: 26594) were identified in PID_108.

PID_15 was found to have single point mutations in three genes of uncertain significance to the DMD phenotype. The variants include a missense heterozygous mutation (c.1655C>G) in exon 1 of the *MAGEL2* gene, a missense heterozygous mutation (c.2234C>A) in the *ZEB2* gene within exon number 8, and another missense heterozygous mutation (c.2188C>T) in the *ABCD1* gene in exon 10. While another individual (PID_16) carried pathogenic mutations in the *DMD* gene and another gene, i.e., a hemizygous frameshift deletion mutation (c.5578delA) in the exon 42 of the *DMD* gene and a heterozygous splicing mutation (c.791+1G>A) in the *LZTR1* gene, respectively (Table 1). CNV analysis from WES data, revealed the duplication of exon 45 and 47 within the *DMD* gene in PID_28, as depicted in Supplementary Fig. 3H.

### Phenotypic heterogeneity between *DMD* mutated and other DMD suspected cases

**Figure 4:**
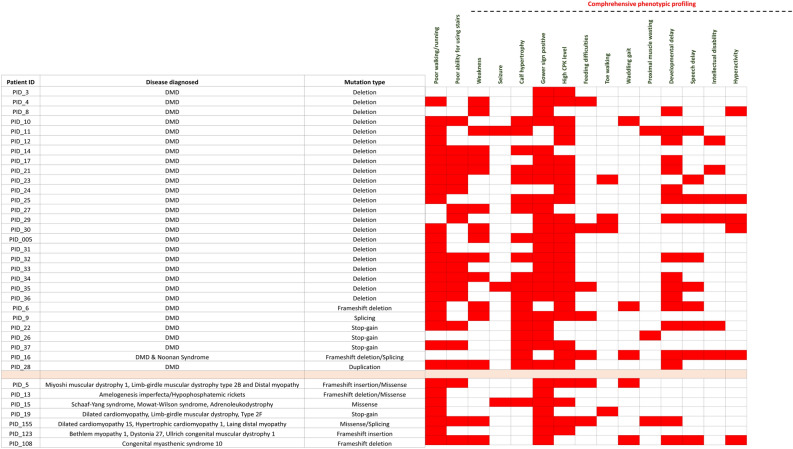
Correlation of phenotypic characteristics between patients who are *DMD* positive and mutated in other non-*DMD* genes. The column represents patient ID, mutated gene, and mutation type. The heatmap shows positive (red square) and absent (white square) phenotypic attributes for each category.

We conducted a clinical correlation analysis between phenotypic characteristics and genetic variants in participants with positive DMD cases who had either exon deletions or point mutations identified through multiplex PCR and exome sequencing as depicted in Fig. 4. Our findings revealed that individuals with *DMD* deletions exhibited a higher incidence of certain phenotypes, including calf hypertrophy and developmental delay, including speech delay, than those with non-*DMD* mutations. Other phenotypes, including poor walking, high CPK, and a positive Gower sign, were consistent across most individuals. Some other phenotypes, such as feeding difficulties and hyperactivity, were seen less frequently and did not segregate with any particular genotype.

## Discussion

DMD is a degenerative, fatal, and incurable muscular dystrophy that is typically diagnosed between the ages of three and four. Affected boys have increasing muscle weakness and cardiorespiratory and orthopedic complications and are at risk of cognitive, behavioral, and language difficulties^30^. Suspected cases often undergo *DMD* gene mutation screening to identify common DMD-associated deletions. Exome sequencing is now becoming a routine test in most developed countries, but it is still not a viable option for most developing countries.

In this study, we conducted a comprehensive analysis of the clinical, pathological, and genetic characteristics of 36 DMD-suspected individuals from Bangladesh to determine genotype–phenotype correlations. We adopted an economically meaningful sequential screening process, where we first screened *DMD* exonic deletion hotspots using multiplex PCR followed by exome sequencing for hotspot negative individuals. To our knowledge, this is the first attempt to combine hotspot screening coupled with exome sequencing to detect *DMD* and other relevant mutations in the Bangladeshi cohort. This diagnostic strategy will facilitate the implementation of national guidelines for DMD-suspected patients in Bangladesh and other developing nations. The higher diagnostic rate might be due to the combine factor of ethnic specific hotspots and the combine diagnostic strategies.

Our findings suggest that screening for *DMD* hotspot deletions can efficiently identify mutations for most of individuals, and exome sequencing can be reserved for those who do not have hotspot deletions. The results of the current study demonstrate that the diagnostic yield achieved by multiplex PCR and WES is 61.1% and 97.2%, respectively. These findings highlight the superior efficacy of WES for identifying mutations compared to multiplex PCR, albeit at a higher cost. WES represents a highly efficient and effective technique as it enables the screening of all genes associated with muscular dystrophies. As we have shown, a small but significant number of people presenting a phenotype compatible with DMD have mutations in genes other than *DMD*. Moreover, previous research in our lab has shown the good diagnostic yield of WES as a first-line diagnostic approach for rare disease patients in developing countries who lack a clear differential diagnosis, and where healthcare resources are limited^31^. Of the 36 individuals studied, 22 (61%) had a positive diagnosis from the *DMD* hotspot deletion test and did not require exome sequencing. Of the 29 DMD-positive individuals, 22 (75.86%) had a deletion mutation, 1 (3.44%) had duplication mutation, 3 (10.3%) had a stop-gain mutation, 2 (6.89%) had a frameshift deletion, and only 1 (3.44%) had a splice-site mutation. In our study, we have expanded the analysis to include a comparative assessment with established DMD cohorts as reported in the literature. Notably, a review of data from DMD cohorts^32–35^ reveals a consistent pattern of genotype–phenotype correlations that align with our findings in Bangladeshi population. For instance, the frequency of deletions and duplications in the *DMD* gene reported by^32^ corresponds closely to the distribution observed in our cohort, where 60.2% of individuals exhibited deletions and 9.6% had duplications^34^. reported 81.2% deletion and 5.4% duplication in *DMD* gene and 1.81% mutations in other genes. This cross-population consistency underscores the potential universality of the mutational spectrum in DMD and its related disorders. Our findings contribute to the broader understanding of DMD's genetic landscape and provide a foundation for exploring the unique aspects of the disease as it presents in the Bangladeshi population.

We found the frequency of multiplex PCR identified exonic deletions in the DMD cases was 61% which is much greater than the reported deletion rates among other Asian populations using the same method, i.e., 40% in Singapore, 51.2% in Japan, 32.4% in Vietnam, 49% in Thailand, and 44.1% in Indonesia^36–38^. Furthermore, our findings indicate that most of the identified deletions in the exons were at the distal end of the gene. In a prior study^39^, the proximal and distal boundaries were categorized as 1–30 and 31–79, respectively. Within our cohort, we observed that 81.8% (18/22) of patients had deletion-type mutations near the distal hotspot, while 13.63% (3/22) of patients exhibited deletion mutations near the proximal end. These data are in accordance with the earlier studies based on multiplex PCR that showed approximately 20–30% of detected dystrophin gene deletions were in the proximal hotspot and ~ 70–80% in the distal hotspot^40^. In our study, we observed deletion patterns that do not always conform to previously reported associations between specific deletions and disease severity. For example, prior research^41^ suggested that deletions near the distal region, spanning exons 45 to 48, were linked to a relatively high level of dystrophin and a mild Becker Muscular Dystrophy (BMD) phenotype. However, in our study, none of the 17 patients (PID_4, PID_8, PID_10, PID_11, PID_12, PID_17, PID_21, PID_23, PID_25, PID_29, PID_30, PID_005, PID_31, PID_32, PID_33, PID_34, & PID_35) with deletions in exons 45–53 exhibited mild BMD phenotypes. Instead, these patients presented with early-onset symptoms typical of Duchenne Muscular Dystrophy (DMD), as detailed in Supplementary Table 3. Similarly, previous studies^42^ have suggested that mutations in the proximal part of the *DMD* gene are generally associated with more severe defects than mutations in the distal part. In our study, three patients (PID_3, PID_14, & PID_24) had deletions near the proximal ends. Interestingly, only two of them exhibited elevated creatine kinase (CK) levels, which were comparatively lower than in others. These discrepancies between our findings and earlier research indicate that patients with similar mutations and protein levels may experience significantly different clinical progressions. This suggests that additional factors, such as epigenetic and environmental influences, play a substantial role in determining the severity of a patient's disease^22^. Moreover, in patient PID_16, as outlined in Table 1, we observed a co-occurrence of mutations in both the *DMD* gene and *LZTR1*, which is associated with Noonan Syndrome. This patient presented with a combination of symptoms, encompassing DMD-like manifestations, such as calf hypertrophy, elevated CPK levels, a waddling gait, and walking difficulties, and additionally symptoms associated with Noonan Syndrome^43^, including developmental delay, speech delay, hyperactivity, autistic traits, and intellectual disability. However, although patient PID_16 exhibited overlapping symptoms, the available data did not definitively categorize them as Noonan syndrome in the absence of particular craniofacial abnormalities.

Interesting point mutations and novel nucleotide sequence variants were observed in our study cohort, including novel pathogenic mutations in the *DMD* gene and novel variants in *SGCD*, *DYSF*, *COL6A3*, and *DOK7* genes. These findings expand the mutational spectrum of muscular dystrophies and provide valuable information for the molecular diagnosis of DMD patients. In patient PID_19, we discovered a novel stop-gain mutation in exon 4 of *SGCD*. Mutations in the *SGCD* gene have been linked to developmental problems in both human and animal models^44^. In PID_5, a novel heterozygous frameshift insertion in *DYSF* was identified, which accounts for muscular dystrophy. A study of 72 Korean people with Miyoshi Muscular Dystrophy (MMD1) or Limb Girdle Muscular Dystrophy type 2B (LGMDR2) found 49 different disease-causing *DYSF* variants^45^. According to the report, the affected individuals displayed a positive Gower sign and walking and stair climbing difficulties.

Additionally, two mutations in *COL6A3* were found in two different individuals. PID_155 had a heterozygous splicing mutation (c.6210+1G>A) in exon 16, and PID_123 had a heterozygous frameshift insertion (c.3958 3959insGTGT) in exon 9. To our knowledge, the mutation in exon 9 is novel and is being reported here for the first time. Reports demonstrate that *COL6A3* mutations cause a wide range of disorders characterized by muscle weakness and connective tissue abnormalities, ranging from severe Ullrich congenital muscular dystrophy to mild Bethlem myopathy^46–48^. We also identified a novel homozygous frameshift deletion (c.1134delG) in exon 7 of the *DOK7* gene mutation in PID_108, reportedly linked to the congenital myasthenic syndrome. Clinical observations showed that this patient displayed a positive Gowers sign, walking difficulties, and a delayed developmental milestone. Studies suggest that the phenotypic presentation of mutations associated with the *DOK7* gene varies significantly across the patients, depending on how protein sequence is affected, therefore, leading to various pathophysiological conditions with an onset age ranging from early adolescence to early adulthood^49–51^. In the case of Patient PID_13, we observed a noteworthy genetic profile. This patient harbored a homozygous frameshift deletion (c.764_768del) in exon 8 of *WDR72*, known to be associated with Amelogenesis imperfecta, type IIA3. Additionally, another homozygous missense mutation (c.10G>C) in exon 1 of *PHEX* was identified, which is typically linked to hypophosphatemic rickets. Initially, Patient PID_13 presented with symptoms including a positive Gower sign and walking difficulties (Supplementary Table 3), leading to suspicion of Duchenne muscular dystrophy, prompting further evaluation. Interestingly, muscle-related symptoms can occasionally manifest in patients with *PHEX* gene mutations^52^. Despite this we are unable to say with certainty whether this gene or *WDR72* contributed to this patient’s muscular phenotype. Subsequent communication with the patient's parents confirmed the presence of tooth discoloration, which aligns with one of the characteristic features of Amelogenesis imperfecta (OMIM# 613211).

From our study, we determined that utilizing multiplex PCR and WES allowed us to identify mutations in the *DMD* gene in 80.5% of cases. Additionally, we conducted CNV analysis for the eight patients who did not exhibit pathogenic variants in the *DMD* gene, as shown in Supplementary Fig. 3. Remarkably, our CNV analysis unveiled a significant finding. Patient PID_28, previously classified as a negative case, was identified as positive for a *DMD* duplication mutation, as illustrated in Supplementary Fig. 3H. This discovery underscores the importance of comprehensive genetic analysis in refining diagnostic accuracy and its potential impact on patient care.

Phenotypic and cellular heterogeneity is very common in rare disorders^7,19,53^. Our study sheds light on the phenotypic diversity of patients with *DMD* mutations. Our phenotypic analysis revealed that patients with *DMD* mutations exhibited a wide range of clinical features. These included poor walking, a positive Gower sign and elevated CPK levels among the majority, and developmental delay, speech delay, and intellectual disability to a variable degree. There was no pattern by whether the mutation was distal or proximal, or type of deletion. To fully realise why some, but not all, DMD individuals have these additional developmental comorbidities will require the accumulation of evidence from larger samples, as well as more detailed genomic analysis. These findings do, of course, highlight the importance of undertaking a detailed neurodevelopmental evaluation such as ours in any child presenting with suspected DMD.

In our study, the identification of novel mutations within DMD population presents a compelling avenue for further investigation. Recognizing the limitations posed by the absence of support studies, we propose several approaches for future research. Functional assays, such as reporter gene assays and protein interaction studies, could provide vital insights into the consequences of these mutations at a molecular level. *In-vitro* studies, including the use of patient-derived iPSCs to model DMD pathology, would further elucidate the impact of novel mutations on disease progression. By integrating these strategies, subsequent studies can aim to translate these initial findings into a more profound understanding of the pathogenic mechanisms of DMD and related disorders, potentially leading to targeted therapeutic interventions.

Our study also identified some variability in clinical presentation that may earmark specific differences between DMD and other, non-DMD dystrophies. These included the observation that non-DMD dystrophies were less likely to present with calf hypertrophy and developmental delay / intellectual disability. These findings could aid in the differential diagnosis of DMD and other neuromuscular disorders. Overall, our study highlights the excellent diagnostic yield of genetic testing and its contribution towards a more personalized treatment approach in the management of DMD and other early onset dystrophies. Our findings have important implications for clinicians working in the field of neuromuscular disorders and could inform the development of new treatment strategies for patients with DMD.

## Conclusions

In conclusion, our study has contributed to mutation mapping in the *DMD* exonic deletion hotspot as well as identifying novel mutations impacting other genes related to neuromuscular disorders. By specifically focussing on the Bangladeshi population, we have provided evidence of the effectiveness of our approach in a developing country as well as furthering our understanding of the mutational landscape and phenotypic variation of DMD and related dystrophies globally. Since there have been limited prior investigations into diseases like DMD in developing nations, our study contributes to the scarce literature on mutations or variants associated with rare disorders in this population^18,31,54^. The identification of mutations associated with the *DMD* gene is critical for accurate illness prognosis, genetic counseling, and personalized genetic therapy. As DMD is a progressive disease that causes irreversible muscle atrophy, early identification allows all potential therapeutic approaches to be considered at an early stage. Our findings in the Bangladeshi study cohort have identified novel pathogenic variants of the *DMD* gene and other phenotype-muscular dystrophy associated genes, thereby expanding the human mutational database and the spectrum of DMD-related pathogenesis. These findings have important implications for improving the clinical management of neuromuscular disorders in this population and beyond.

### Supplementary Information

Supplementary Tables.

Supplementary Figures.

## Data Availability

Participant consent does not allow the sharing of any additional phenotype or genomic information beyond that contained in this manuscript. However, other data are available upon reasonable request to corresponding authors for replication or extensions of this study.
