# Supplementary material for: Mutational spectrum and phenotypic variability of Duchenne muscular dystrophy and related disorders in a Bangladeshi population
Source: Sci Rep. 2023 Dec 6;13:21547. doi: 10.1038/s41598-023-48982-w (PMC10700514; doi:10.1038/s41598-023-48982-w)
Supplement: Supplementary file 2 — Supplementary Figures. [file 41598_2023_48982_MOESM2_ESM.pdf]

(A)

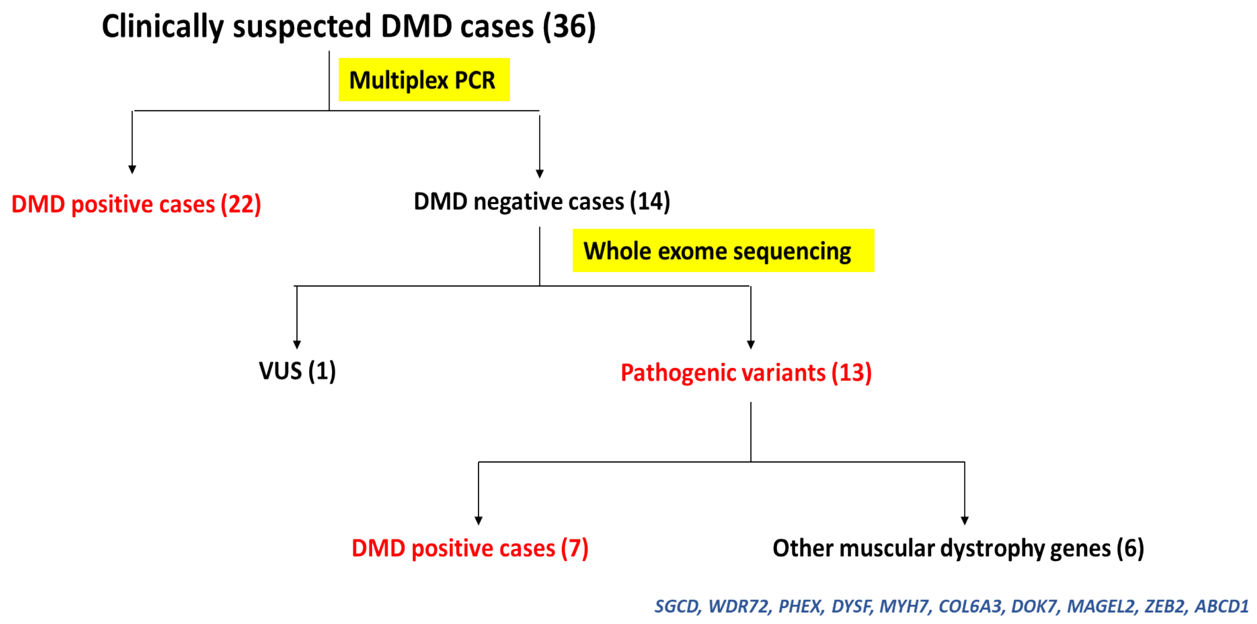

(B)

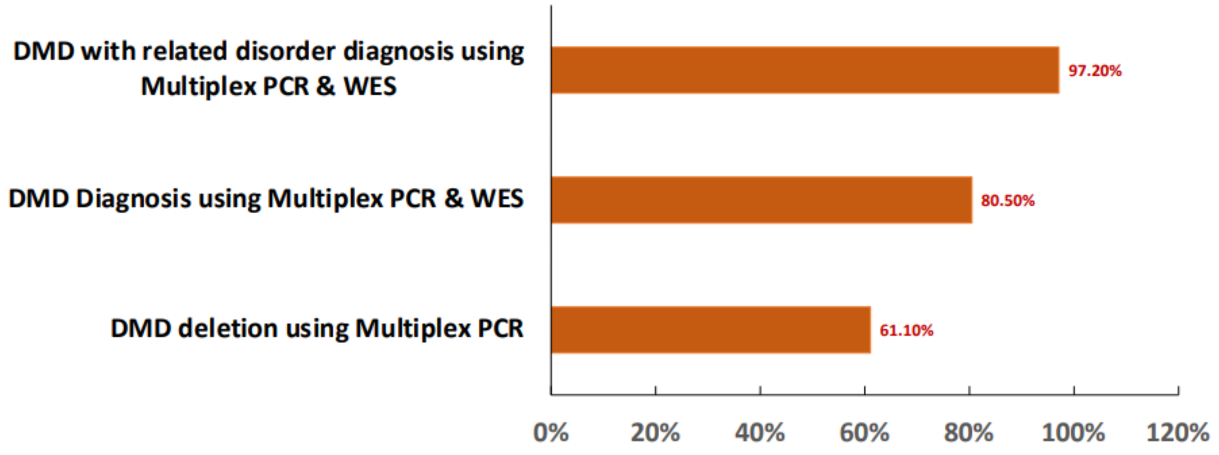

Supplementary Figure 1: (A) Schematic diagram showing the step-by-step process of our genetic screening. The yellow boxes represent the technologies and red texts indicate the *DMD* positive cases. (B) Diagnostic yield obtained from the DMD-suspected cohort using two technologies multiplex PCR and whole exome sequencing.

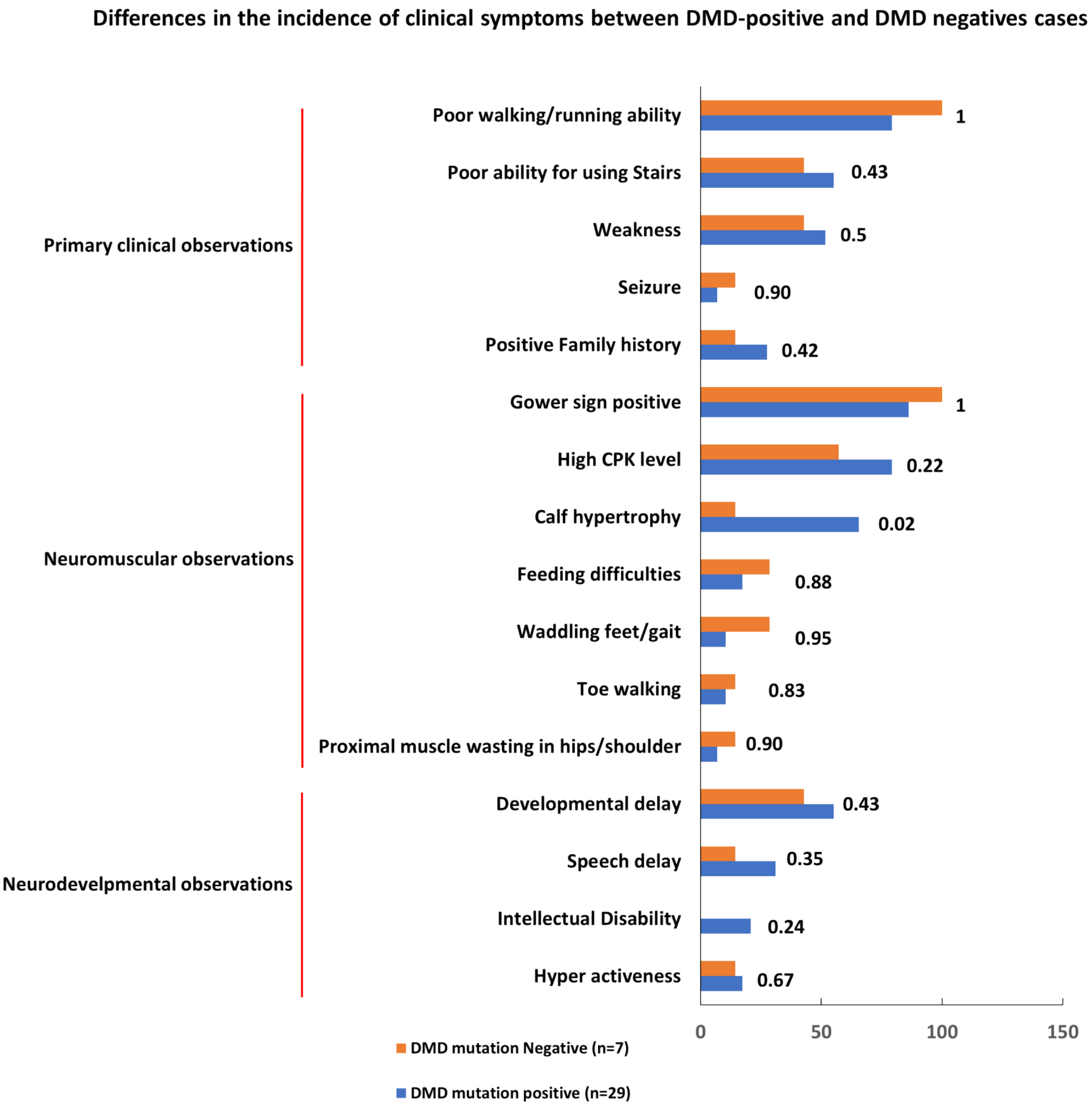

Supplementary figure 2: The variations in the occurrence of specific clinical symptoms between *DMD* mutation-positive and *DMD* mutation-negative cases has been analyzed and the p-values are computed using the Fisher exact test. It was observed that only the difference in the phenotype 'Calf hypertrophy' between *DMD* mutation-positive and *DMD* mutation-negative cases is significant (p= 0.02).

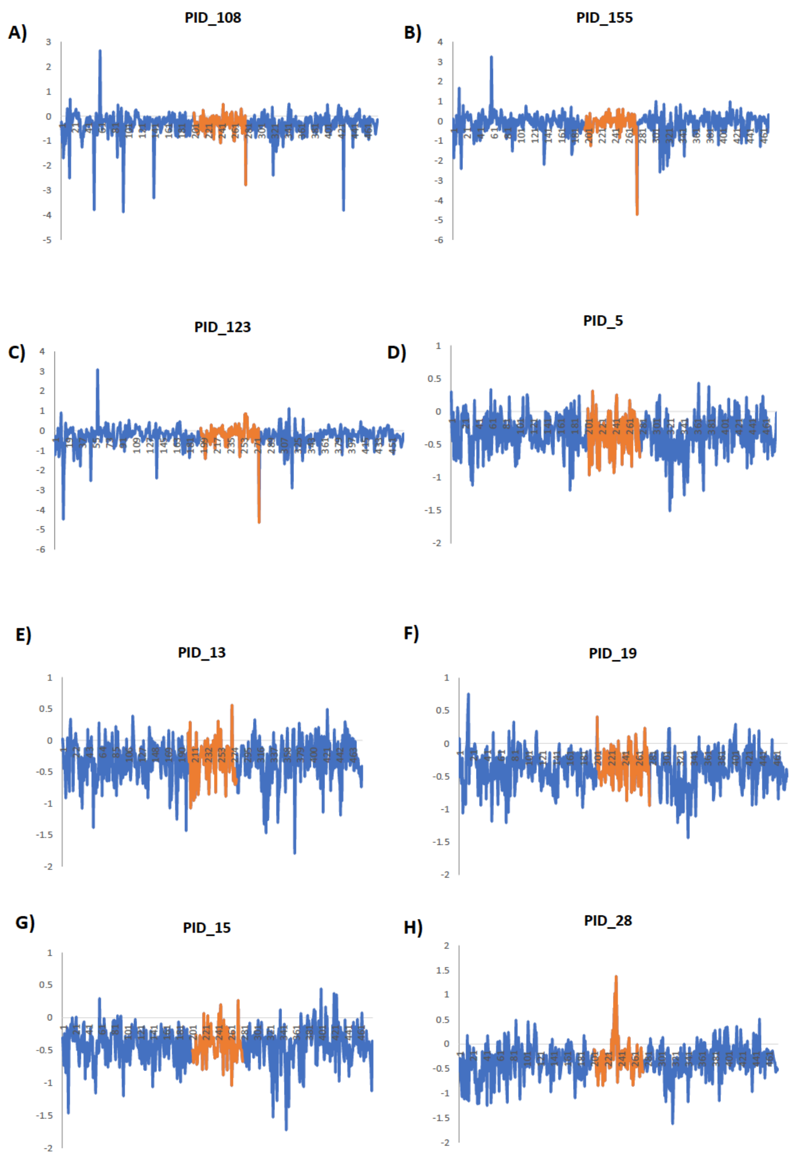

Supplementary Figure 3: Copy Number Variation Analysis Using CNVkit on WES Data. The orange line represents the region of *DMD* gene (log scale y-axis). Duplication analysis for 8 DMD patients (A-H) reveal only one event (H) crossing the significant threshold.
